# Clotrimazole Drug Resistance in *Candida glabrata* Clinical Isolates Correlates with Increased Expression of the Drug:H^+^ Antiporters CgAqr1, CgTpo1_1, CgTpo3, and CgQdr2

**DOI:** 10.3389/fmicb.2016.00526

**Published:** 2016-04-19

**Authors:** Catarina Costa, Jonathan Ribeiro, Isabel M. Miranda, Ana Silva-Dias, Mafalda Cavalheiro, Sofia Costa-de-Oliveira, Acácio G. Rodrigues, Miguel C. Teixeira

**Affiliations:** ^1^Department of Bioengineering, Instituto Superior Técnico, University of LisbonLisboa, Portugal; ^2^Institute for Bioengineering and Biosciences, Biological Sciences Research GroupLisboa, Portugal; ^3^Department of Microbiology, Faculty of Medicine, University of PortoPorto, Portugal; ^4^CINTESIS-Center for Health Technology and Services Research, Faculty of Medicine, University of PortoPorto, Portugal

**Keywords:** azole drug resistance, clotrimazole, fluconazole, drug:H^+^ antiporters, clinical isolates, *Candida glabrata*

## Abstract

For years, antifungal drug resistance in *Candida* species has been associated to the expression of ATP-Binding Cassette (ABC) multidrug transporters. More recently, a few drug eﬄux pumps from the Drug:H^+^ Antiporter (DHA) family have also been shown to play a role in this process, although to date only the *Candida albicans* Mdr1 transporter has been demonstrated to be relevant in the clinical acquisition of antifungal drug resistance. This work provides evidence to suggest the involvement of the *C. glabrata* DHA transporters CgAqr1, CgQdr2, CgTpo1_1, and CgTpo3 in the clinical acquisition of clotrimazole drug resistance. A screening for azole drug resistance in 138 *C. glabrata* clinical isolates, from patients attending two major Hospitals in Portugal, was performed. Based on this screening, 10 clotrimazole susceptible and 10 clotrimazole resistant isolates were selected for further analysis. The transcript levels of *CgAQR1*, *CgQDR2*, *CgTPO1_1*, and *CgTPO3* were found to be significantly up-regulated in resistant isolates when compared to the susceptible ones, with a level of correlation that was found to be similar to that of *CgCDR2*, an ABC gene known to be involved in the clinical acquisition of resistance. As a proof-of-concept experiment, the *CgTPO3* gene was deleted in an azole resistant *C. glabrata* isolate, exhibiting high levels of expression of this gene. The deletion of *CgTPO3* in this isolate was found to lead to decreased resistance to clotrimazole and fluconazole, and increased accumulation of azole drugs, thus suggesting the involvement of this transporter in the manifestation of azole resistance.

## Introduction

In recent years, *Candida glabrata* has become the second most common cause of mucosal and invasive candidosis, only surpassed by *C. albicans* (Vermitsky and Edlind, 2004; Rodrigues et al., 2014; Yapar, 2014). *C. glabrata* is, indeed, responsible for 15–20% of all known *Candida* infections, with its relative incidence increasing every year (Roetzer et al., 2011). The extensive use of antifungal drugs both as treatment and prophylaxis has led to a huge increase in the number of intrinsically resistant infections with fungal pathogens. The frequency and relative high mortality levels of these infections are generally attributed to the capacity of these pathogenic yeasts to efficiently develop multiple drug resistance (MDR; Vermitsky and Edlind, 2004; Sanguinetti et al., 2005; Rezaei et al., 2009).

Azole antifungals, including fluconazole (FLC) and clotrimazole (CLT), are commonly used in clinical practice (Pina-Vaz et al., 2001; Vermitsky and Edlind, 2004). One of the most frequent azole formulations used for treatment of fungal mucocutaneous infections, such as vaginal and oropharyngeal candidiasis, is the imidazole clotrimazole (Crowley and Gallagher, 2014). Fluconazole, on the other hand, has been extensively used in prophylaxis and in the therapy of candidosis in organ and bone marrow transplant recipients, patients undergoing chemotherapy and AIDS patients. It has been shown that prolonged fluconazole exposure may favor the up rise of *C. glabrata* infections (Abbes et al., 2013; Papon et al., 2013). Both drugs act by inhibiting lanosterol 14α-demethylase, product of the *ERG11* gene (Vermitsky and Edlind, 2004), a key enzyme in ergosterol biosynthesis. However, a disturbing percentage of the *C. glabrata* clinical isolates have been shown to display azole resistance, unlike what has been observed for most other *Candida* species (Sanguinetti et al., 2005). For example, in a study involving 33 *C. glabrata* isolates, 20 were found to be resistant to fluconazole and ketoconazole (Sanguinetti et al., 2005).

There are mainly three described mechanisms of azole resistance in *Candida* spp.: (i) increased production of the azole target enzyme Erg11, (ii) point mutations in the same enzyme, affecting drug binding, and (iii) drug eﬄux mediated by Cdr1 and Cdr2, multidrug eﬄux pumps belonging to the ATP-binding cassette superfamily (ABC), or, in *C. albicans*, by Mdr1, a major facilitator superfamily (MFS) transporter (Henry et al., 2000; Silva et al., 2011). Additionally, point mutations in *ERG3*, encoding for Δ^5,6^-desaturase, impairing enzyme activity have been described to confer azole resistance (Martel et al., 2010). Recent analysis, however, of the mechanisms underlying the acquisition of azole drug resistance in *C. glabrata* suggest that the expression level or amino acid substitutions of the *ERG11* gene do not seem to correlate with the azole resistance levels in this fungal pathogen (Sanguinetti et al., 2005; Szweda et al., 2015).

In *C. glabrata*, azole resistance has also been found to be closely related with the action of the ABC superfamily multidrug transporters CgCdr1 and CgCdr2 (Sanguinetti et al., 2005; Silva et al., 2011). MDR transporters belonging to the MFS have recently been associated to this phenomenon (Costa et al., 2013a,b, 2014b). CgAqr1 has been shown to confer resistance to flucytosine and, to a lower extent, clotrimazole, (Costa et al., 2013a), while CgQdr2 was found to confer resistance to the imidazoles clotrimazole, thioconazole, miconazole, and ketoconazole (Costa et al., 2013b), and CgTpo1_1, CgTpo1_2 and CgTpo3 were shown to confer resistance to imidazoles as well as to the triazoles itraconazole and fluconazole (Costa et al., 2014b; Pais et al., 2016). As for the remaining five predicted drug transporters of the same family in *C. glabrata*, only CgFlr1 was characterized (Costa et al., 2014a), but shown not to be involved in azole drug resistance (Chen et al., 2007). However, their clinical relevance had yet to be assessed as, for instance, in *C. albicans*, Mdr1 has been consistently found to be overexpressed in fluconazole resistant isolates (Wirsching et al., 2000a,b), but that was not the case of *C. albicans* Flu1 transporter.

In this study, the clinical relevance of the five DHA transporters previously found to be involved in azole resistance in *C. glabrata* (CgAqr1, CgQdr2, CgTpo1_1, CgTpo1_2, and CgTpo3) was evaluated. A collection of clinical isolates was characterized in what concerns susceptibility to two drugs, representative of azole antifungal drug families: fluconazole, a triazole used in the treatment and prophylaxis of invasive candidiasis, and clotrimazole, an imidazole used to treat mucosal infections. In strains selected for displaying high or low clotrimazole MIC values the expression of *CgAQR1*, *CgQDR2*, *CgTPO1_1*, *CgTPO1_2*, and *CgTPO3* was analyzed, and compared to that of *CgCDR1* and *CgCDR2*, used as positive controls. Furthermore, one of the DHA genes under analysis, *CgTPO3* was deleted in an azole resistant clinical isolate, exhibiting high levels of *CgTPO3* expression, and its effect on azole susceptibility was analyzed.

## Materials and Methods

### *Candida glabrata* Strains and Growth Media

Two collections of clinical strains, comprising 75 isolates collected from patients admitted to Hospital of Santa Maria (HSM), Lisboa, and 63 isolates harvested from patients attending Centro Hospitalar São João (CHSJ) (Costa-de-Oliveira et al., 2008; Faria-Ramos et al., 2014), Porto, were used in this study (Supplementary Table S1). Cells were batch-cultured at 30°C with orbital agitation (250 rpm) in YPD growth media, with the following composition (per liter): 20 g glucose (Merck), 20 g yeast extract (Difco) and 10 g peptone (Difco). For some of the experiments basal medium (BM) was used with the following composition (per liter): 1.7 g yeast nitrogen base without amino acids or NH_4_^+^ (Difco), 20 g glucose (Merck) and 2.65 g (NH_4_)_2_SO_4_ (Merck). Solid media contained 20 g/L agar (Iberagar) besides the ingredients listed above. Fluconazole (FLC) and Clotrimazole (CLT) were obtained from Sigma, prepared in dimethyl sulfoxide (Sigma), frozen at -80°C until use. The antifungal solutions used for the MIC determination were diluted with RPMI 1640 medium (Sigma) and buffered to pH 7.0 with 0.165 M morpholinepropanesulfonic acid buffer (Sigma).

### Susceptibility Testing of *C. glabrata* Isolates

The Minimal Inhibitory Concentration (MIC) of each antifungal drug was determined according to the M27-S4 protocol of the Clinical and Laboratory Standards Institute (CLSI) (CLSI, 2008, 2012), for the isolates obtained from Centro Hospitalar São João, and by the EUCAST definitive document EDef 7.2 (Rodríguez-Tudela et al., 2003), for the isolates collected at Hospital of Santa Maria. EUCAST and CLSI methods have been shown to provide similar results in terms of MIC determinations (Cuenca-Estrella et al., 2002; Rodriguez-Tudela et al., 2007; Pfaller et al., 2011). The MICs were determined after 48 h of incubation for both FLC and CLT. The susceptibility breakpoints for FLC were those proposed by the CLSI M27-S4 document. For FLC, the MIC value thresholds to consider a strain susceptible-dose dependent or resistant were ≤32 μg/ml or ≥64 μg/ml, (CLSI, 2008, 2012). In the case of CLT there are no established breakpoints. The considered MIC value thresholds – for susceptibility ≤0.5 μg/ml and for resistance ≥1 μg/ml – were according to previously published standards for *C. albicans* (Pelletier et al., 2000). The *C. glabrata* CBS138 type strain was used for quality control of antifungal susceptibility testing.

The susceptibility of the clinical isolate 51800, and of the derived 51800_Δ*tpo3* mutant, to CLT and FLC was compared based on MIC determination, as described above, and on growth in solid medium. *C. glabrata* cells were grown in BM medium until mid-exponential phase (OD_600nm_ = 0.4 ± 0.02) and diluted in sterile water to obtain suspensions with OD_600nm_ = 0.05 ± 0.005. These cell suspensions and subsequent dilutions (1:5 and 1:25) were applied as 4 μl spots onto the surface of agarized BM medium, supplemented with 20 and 24 mg/l of CLT and 475 and 500 mg/L of FLC.

### Gene Expression Analysis

Ten CLT susceptible and 10 CLT resistant isolates were selected (**Table 3**) to assess the transcript levels of the MFS-MDR genes *CgAQR1*, *CgQDR2*, *CgTPO1_1*, *CgTPO1_2*, and *CgTPO3* and of the ABC genes *CgCDR1* and *CgCDR2* by quantitative real-time PCR (qRT-PCR). Total-RNA samples were obtained from cell suspensions harvested upon reaching an OD_600nm_ = 0.8 ± 0.08 (mid-exponential-phase cells). cDNA for real-time reverse transcription-PCR was synthesized from total-RNA samples by using the MultiScribe^TM^ reverse transcriptase kit (Applied Biosystems) and the 7500 RT-PCR thermal cycler block (Applied Biosystems). The quantity of cDNA for subsequent reactions was kept at ca. 10 ng. The subsequent RT-PCR step was carried out using SYBR green reagents. Primers for the amplification of the five genes and *CgACT1* were designed using Primer Express software (Applied Biosystems) and are summarized in **Table 1**. The RT-PCR was carried out using a thermal cycler block (7500 real-time PCR system; Applied Biosystems). Default parameters established by the manufacturer were used, and fluorescence was detected by the instrument and recorded in an amplification plot (7500 System SDS software; Applied Biosystems). The *CgACT1* mRNA level was used as an internal control. The relative values obtained for the clotrimazole-susceptible isolate exhibiting the lower gene expression level were set as 1 and the remaining values are presented relative to that control. Statistical analysis of the results was performed using ANOVA, and differences were considered statistically significant for *p*-values < 0.05.

**Table 1:** Pairs of primers designed for RT-PCR.

Gene	Primer	Sequence
*CgACT1*	Forward	5′ -AGAGCCGTCTTCCCTTCCAT- 3′
	Reverse	5′ -TTGACCCATACCGACCATGA- 3′
*CgAQR1*	Forward	5′ -GCTGATAAGTTCGGCCGTAGA -3′
	Reverse	5′ -AATGGAGGCAACCACGTAGATC- 3′
*CgCDR1*	Forward	5′ -GCTTGCCCGCACATTGA -3′
	Reverse	5′ -CCTCAGGCAGAGTGTGTTCTTTC- 3′
*CgCDR2*	Forward	5′ -GCCATGGTACCTGCATCGAT- 3′
	Reverse	5′ -CCGAGGAATAGCAAAACCAGTATAC- 3′
*CgQDR2*	Forward	5′ -TCACTGCATAGTTTCATATCGGACTA- 3′
	Reverse	5′ -CAACTTCAGATAGATCAGGACCATCA- 3′
*CgTPO1_1*	Forward	5′ -CGCTGCTTCCCCAGTTATCT- 3′
	Reverse	5′ -CTAGCACACCACGTCTACCGTAA- 3′
*CgTPO1_2*	Forward	5′ -AGGACCCGCTCTATCGAAAAA- 3′
	Reverse	5′ -GCTGCGACTGCTGACTCAAC- 3′
*CgTPO3*	Forward	5′ -TGCCGATATGTTCCCAAGTGA- 3′
	Reverse	5′ -TGGAGCGAAAGCGAAGAAAG- 3′

### Disruption of *CgTPO3*

The deletion of gene *CgTPO3* was carried out in the parental strain 51800 using the method described by Reuss et al. (2004). The gene to be deleted was replaced by a *SAT1* flipper cassette by homologous recombination using primers 5′ –CCCTCCAATCCAGATTGACGCAGTGGGGTTATAGGTTACTGAGGTGTTTCTATATATACA*ATGGACGGTGGTATGTTT*- 3′ and 5′ –ATATATTATGATTCAATGAGAAGTACATTAGATGTAGGAGGTGGAAGTAAGGGGAGTTGT*TTAGGCGTCATCCTGTGCTC*- 3′. The underlined region of the primers has homology with the gene to be amplified while the italic region has homology with the *SAT1* flipper cassette encoding sequence. The pA83 plasmid including *CgSAT1* was used as a template, and transformation was performed using the Alkali-Cation Yeast Transformation Kit (MP Biomedicals). Appropriate PCR products were identified and verified by PCR using the following pairs of primers: 5′ –CAGAATTTGAACCTTCGGTG- 3′ which is assigned to the inside of the open reading frame of *CgTPO3*, and 5′ –GCCCAGATAACAACACAAGTCC- 3′ which is specific for the cassette DNA. No PCR products were identified from the template DNA of the mutant, while a clear PCR product was identified from the template DNA of the parental strain.

### [^3^H]-Clotrimazole Transport Assays

[^3^H]-clotrimazole accumulation assays were carried out as described before (Costa et al., 2013b). To estimate its accumulation (Intracellular/Extracellular) in yeast cells, they were grown in BM medium till mid-exponential phase, harvested, washed and resuspended in BM medium, to obtain cell suspensions with an OD600nm = 5.0 ± 0.1, equivalent to approximately 2.2 mg (dry weight) ml^-1^. After 5 min thermostatization at 30°C, 0.1 μM of ^3^H-clotrimazole (American Radiolabeled Chemicals; 1 mCi/ml) and 30 mg/l of unlabeled clotrimazole were added to the suspension. The accumulation of ^3^H-clotrimazole, followed for 30 min, was followed by filtering 200 μl of cell suspension, at adequate time intervals, through glass microfiber filters (Whatman GF/C). The filters were washed with ice-cold TM buffer and the radioactivity measured in a Beckman LS 5000TD scintillation counter. Extracellular concentration of ^3^H-clotrimazole was estimated, by radioactivity assessment of 50 μl of the supernatant. To calculate the intracellular concentration of each radiolabeled compound, the internal cell volume (Vi) of the exponential cells, grown in the absence of drug and used for accumulation assays, was considered constant and equal to 2.5 μl (mg dry weight)^-1^ (Rosa and Sa-Correia, 1996). Statistical analysis of the results was performed using ANOVA, and differences were considered statistically significant for *p*-values < 0.05.

## Results

### Susceptibility Profiles of *C. glabrata* Isolates Toward Clotrimazole and Fluconazole

In this study, 138 *C. glabrata* isolates, coming from two major Portuguese hospitals, were screened for azole drug resistance. The majority of these isolates were harvested from women (64%) and from individuals over 65 years (54%). The most common niches from which they were recovered were urine (31%), blood culture (19%), mucus (16%) and in lower number from vaginal smear, bronchoalveolar lavage, pus, feces, and peritoneal fluid (Supplementary Table S1). FLC resistance levels for 29% of this collection, corresponding to the O and OL isolates harvested from Centro Hospitalar S. João (CHSJ), had been previously obtained (Costa-de-Oliveira et al., 2008, 2011; Faria-Ramos et al., 2014).

**FIGURE 1:**
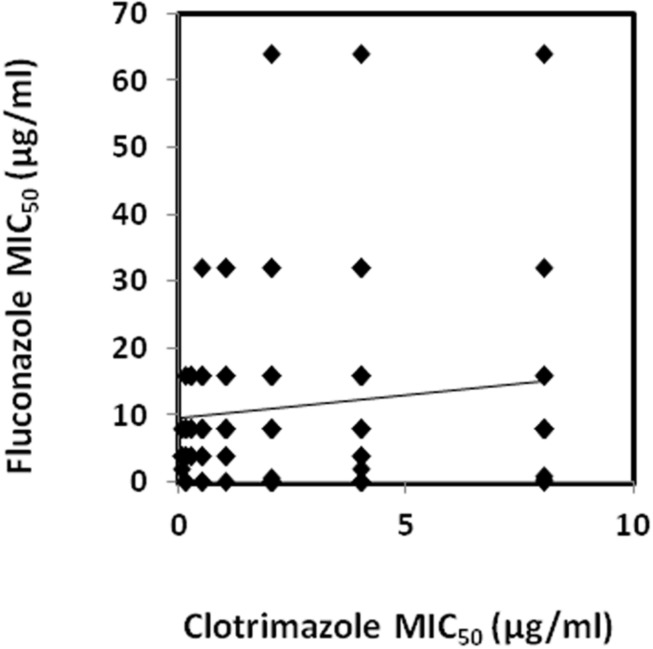
**Correlation between the fluconazole and clotrimazole MIC_50_ values determined for the collection of 138 *Candida glabrata* clinical isolates**. A trendline obtained by linear regression of the correlation values is also displayed. A significant correlation using a Spearman test (0.27) was found to exist between clotrimazole (CLT) and fluconazole (FLC) datasets.

Susceptibility profiles against the CLT and FLC for all *C. glabrata* isolates are summarized in **Table 2** (see details in Supplementary Table S1). Regarding the MIC_50_ values, most isolates (93.5%) were found to be susceptible-dose dependent to FLC, while 9 (6.5%) were found to be resistant to FLC. Additionally, most isolates (64.5%) were found to be resistant to CLT. Interestingly, 48 isolates (34.78%) were found to be resistant to CLT and, at the same time, susceptible-dose dependent to FLC. The opposite, however, was not observed for any of the tested strains. In what concerns CLT, 53.3% of the isolates coming from Hospital of Santa Maria (HSM) were found to be resistant, while isolates harvested in CHSJ had a much higher incidence of clotrimazole-resistance (77.8%). Fluconazole-resistant isolates had a higher incidence in HSM (10.7% vs. 1.6%). Interestingly, using a Spearman test (Spearman coefficient: 0.27) a significant correlation was found to exist between the increase in CLT MIC levels and the FLC MIC levels, when considering the full 138 clinical isolates (**Figure 1**). The level of correlation, however, is relatively low, accounting for a number of isolates exhibiting CLT resistance and very low FLC MIC values, and suggesting that there may be differences in the molecular mechanisms of acquisition of resistance to these related azole drugs.

**Table 2:** Susceptibility profiles of the 138 *Candida glabrata* isolates to the widely used antifungal drugs clotrimazole and fluconazole by the reference broth microdilution method^∗^.

Species	Antifungal	MIC range (Mg/mL)	Origin (no. of isolates)	% of isolates		
				*S*	SDD	*R*
*Candida glabrata*	Fluconazole	0.125–>64	Both hospitals (138)		93.5	6.5
			HSM (75)		89.3	10.7
			CHSJ (63)		98.4	1.6
	Clotrimazole	0.03125-8	Both hospitals (138)	35.5		64.5
			HSM (75)	46.7		53.3
			CHSJ (63)	22.2		77.8

*^∗^Performed as described by Eucast document EDef 7.2 (Rodríguez-Tudela et al., 2003) or CLSI document M27-A3 (CLSI, 2008, 2012)*.

The percentage of clotrimazole- and fluconazole- resistant isolates was found to be similar in both superficial infections and blood infections with 63% of resistant isolates in blood infections vs. 64.9% of resistant isolates in superficial infections for clotrimazole, and 7.4% resistant isolates in blood infections and 6.3% isolates resistant in superficial infections for fluconazole.

### Clotrimazole Resistance in *C. glabrata* Clinical Isolates Correlates with the Expression of the DHA Genes *CgAQR1*, *CgQDR2*, *CgTPO1_1*, and *CgTPO3*

In order to evaluate the predicted role of the Drug:H^+^ Antiporters CgAqr1, CgQdr2, CgTpo1_1, CgTpo1_2, and CgTpo3 in the clinical acquisition of azole drug resistance (Costa et al., 2014a), their expression levels in 20 clinical isolates, 10 exhibiting clotrimazole resistance and 10 exhibiting clotrimazole susceptibility (**Table 3**), were assessed. The option to consider clotrimazole R or S strains for further analysis, and not fluconazole R/SDD strains, is due to the fact that all of the drug transporters under analysis in this study confer imidazole resistance, but only three confer triazole resistance as well. The chosen isolates were also selected for exhibiting the most extreme MIC values. The expression levels of the ABC multidrug eﬄux pump encoding genes *CgCDR1* and *CgCDR2* was also determined, for the sake of comparison, as their over-expression is commonly accepted as a key factor in the clinical acquisition of azole drug resistance (Miyazaki et al., 1998; Sanglard et al., 1999).

**Table 3:** List of the clotrimazole susceptible and resistant *C. glabrata* clinical isolates selected for the determination of the expression levels of the DHA genes *CgAQR1*, *CgQDR2*, *CgTPO1_1*, *CgTPO1_2*, and *CgTPO3*, and of the ABC genes *CgCDR1* and *CgCDR2*.

Susceptible isolates	MIC value (μg/mL)		Resistant isolates	MIC value (μg/mL)	
	CLT	FLC		CLT	FLC
21461	0.0625	4	10774	4	>64
43321	0.03125	4	65147	4	64
48241	0.0625	4	90836	4	32
53351	0.0625	4	94078	4	>64
98495	0.0625	4	51800	8	>64
MC426	0.125	1	O79	8	8
OL090	0.25	8	O155	8	32
MC123	0.5	0.25	MC127	8	0.25
MC125	0.5	0.125	MC165	8	8
MC126	0.5	0.125	MC166	8	16

*Information about fluconazole MIC values for these isolates is also provided*.

**FIGURE 2:**
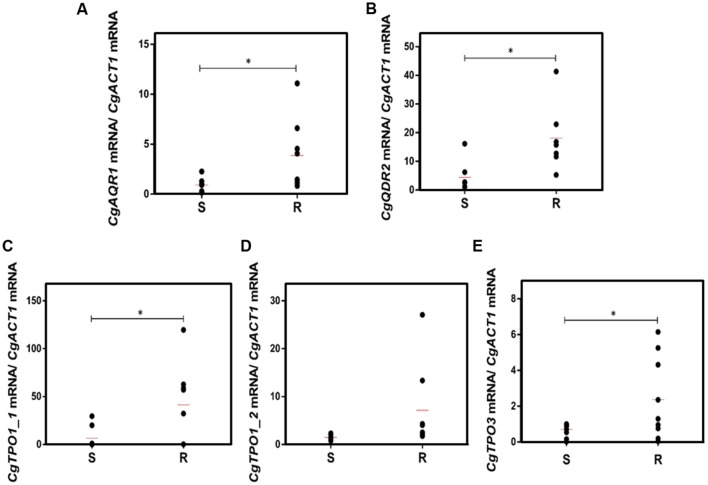
**Transcript levels of **(A)***CgAQR1*, **(B)***CgQDR2*, **(C)***CgTPO1_1*, **(D)***CgTpo1_2*, and **(E)***CgTPO3* in both susceptible (S) and resistant (R) *C. glabrata* clinical isolates**. Transcript levels were assessed through quantitative RT-PCR, as described in the section “Materials and Methods.” The obtained values are the average of at least three independent experiments. The average of the expression values in each group of clinical isolates is represented by a red line (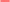). ^∗^*p*-value < 0.05.

**FIGURE 3:**
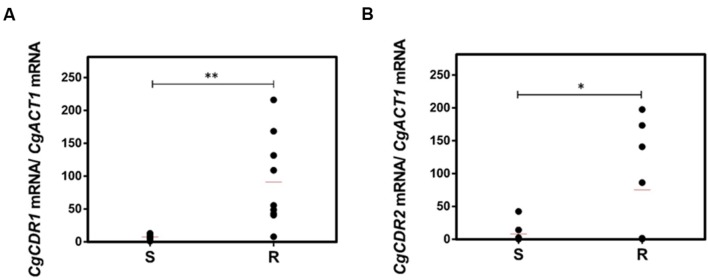
**Transcript levels of **(A)***CgCDR1* and **(B)***CgCDR2* in both susceptible (S) and resistant (R) *C. glabrata* clinical isolates**. Transcript levels were assessed through quantitative RT-PCR, as described in the section “Materials and Methods.” The obtained values are the average of at least three independent experiments. The average of the expression values in each group of clinical isolates is represented by a red line (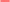). ^∗^*p*-value < 0.05, ^∗∗^*p*-value < 0.01.

A much higher variability in terms of the DHA gene expression was found in the azole-resistant strains, when compared to the susceptible ones. Despite this variability, the transcript level of *CgAQR1, CgQDR2*, *CgTPO1_1*, and *CgTPO3* genes was found to be significantly higher in resistant isolates, when compared to the susceptible ones, considering more than 70% of the tested strains (**Figures 2A–C,E**). In the case of *CgTPO1_2*, no statistically significant correlation could be observed (**Figure 2D**). As expected (Miyazaki et al., 1998; Sanglard et al., 1999; Bennett et al., 2004), the expression of *CgCDR1* and *CgCDR2* was found to correlate with azole drug resistance in the clinical isolates (**Figure 3**). It is important to notice that, the level of correlation between the expression of *CgCDR2* and azole drug resistance was found to be similar to that observed for the DHA genes (*p*-value < 0.05). Consistent with a more relevant role in this context, a higher degree of correlation between gene expression and azole drug resistance was found for *CgCDR1* (*p*-value < 0.01).

### *CgTPO3* Expression Contributes to Azole Resistance in a Resistant Clinical Isolate

**FIGURE 4:**
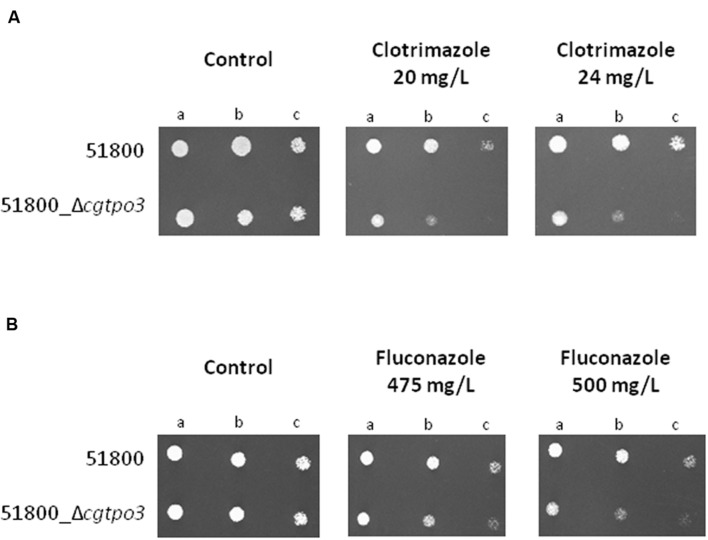
**Comparison of the susceptibility to **(A)** clotrimazole and **(B)** fluconazole, at the indicated concentrations displayed by the clinical isolate 51800 and the derived 51800_Δ*cgtpo3* deletion mutant, in BM agar plates by spot assays**. Cell suspensions used to prepare the spots were (b) 1:5 and (c) 1:25 of the cell suspension used in (a). The displayed images are representative of at least three independent experiments.

*CgTPO3* had previously been shown to contribute to resistance to clotrimazole and fluconazole in a laboratorial strain (Costa et al., 2014b). Given the observation that this gene was found to be up-regulated in clotrimazole resistant clinical isolates, when compared to susceptible ones, it seemed important to assess if its absence could affect azole resistance in clinical isolates as well. Therefore, this gene was deleted in the azole-resistant isolate 51800, found to exhibit high levels of *CgTPO3* expression. The deletion of this gene in the 51800 isolate was found to significantly decrease its resistance to clotrimazole and fluconazole (**Figures 4A,B**), thus reinforcing the notion that this transporter contributes to azole resistance in the clinical context. The MIC_50_ values of clotrimazole and fluconazole for the 51800 and 51800_Δ*cgtpo3* were further evaluated. MIC_50_ values were found to be in all cases higher in the parental 51800 strain (8 and >128 for clotrimazole and fluconazole, respectively) than for the derived Δ*cgtpo3* deletion mutant (4 and 64, respectively).

**FIGURE 5:**
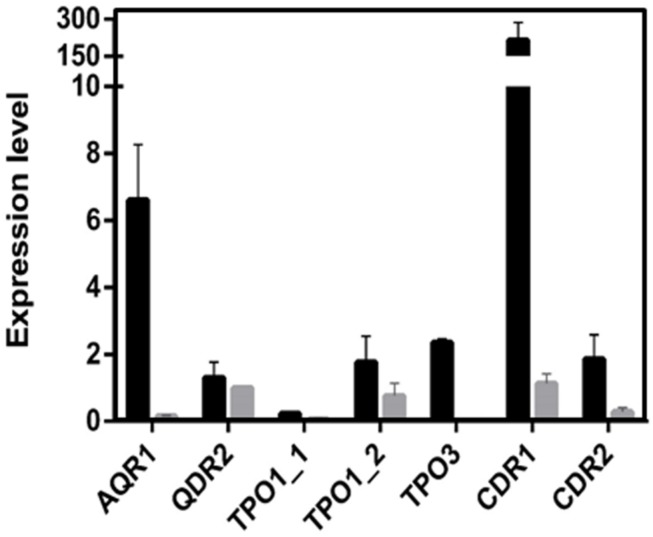
**Transcript levels of *CgAQR1*, *CgQDR2*, *CgTPO1*_*1*, *CgTPO1_2*, *CgTPO3*, *CgCDR1*, and *CgCDR2* in the *C. glabrata* clinical isolate 51800, when compared to those determined in the susceptible isolate exhibiting the lowest level of expression for each of these genes**. Transcript levels were assessed through quantitative RT-PCR, as described in the section “Materials and Methods.” The obtained values are the average of at least three independent experiments. Error bars represent the corresponding standard deviation.

Based on the obtained susceptibility results, it appears clear that CgTpo3 is a player in clinical drug resistance acquisition, but is not fully responsible for the resistance to clotrimazole and fluconazole in the 51800 isolate. Indeed, the expression of additional multidrug transporter encoding genes in this strain was found to be quite high, when compared to what was determined in azole susceptible dose-dependent isolates (**Figure 5**). This is particularly the case for the *CDR1* and *AQR1* genes (**Figure 4**), highlighting the multifactorial nature of azole drug resistance acquisition in the clinical setting.

### *CgTPO3* Mediates ^3^H-Clotrimazole Eﬄux in Clotrimazole-Resistant Isolate 51800

**FIGURE 6:**
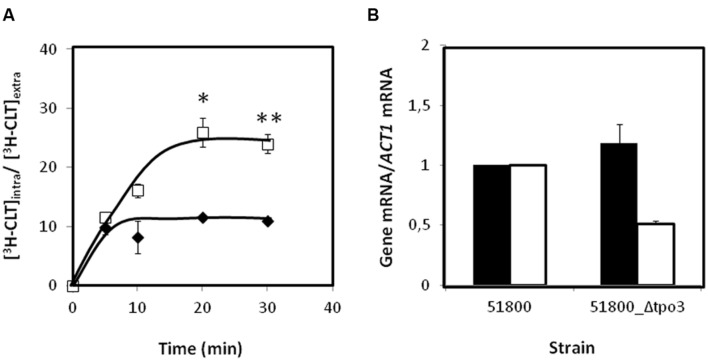
**(A)** Time course accumulation ratio of [^3^H]-Clotrimazole in non-adapted cells of the parental strain 51800 (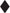) or the mutant strain 51800_*Δcgtpo3* (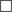), during cultivation in BM liquid medium in the presence of 30 mg/L unlabeled clotrimazole. **(B)** Transcript levels of *CgCDR1* (in black) and *CgCDR2* (in white) in the *C. glabrata* clinical isolate 51800, when compared to those determined in the 51800_*Δcgtpo3* derived deletion mutant. Transcript levels were assessed through quantitative RT-PCR, as described in the section “Materials and Methods.” The obtained values are the average of at least three independent experiments. Error bars represent the corresponding standard deviations. ^∗^*p*-value < 0.05, ^∗∗^*p*-value < 0.01.

Given this observation and the previous demonstration that *CgTPO3* mediates ^3^H-clotrimazole eﬄux in *C. glabrata* (Costa et al., 2014b), CgTpo3 ability to reduce the accumulation of radiolabeled clotrimazole in the *C. glabrata* clinical isolate 51800 was evaluated. The accumulation of ^3^H-labeled clotrimazole in non-adapted *C. glabrata* 51800 and 51800_Δ*cgtpo3* cells, suddenly exposed to the presence of 30 mg/L of cold clotrimazole, was assessed (**Figure 6A**). In these conditions, cells devoid of *CgTPO3* accumulate twofold higher levels of ^3^H-clotrimazole when compared to the parental strain. Since it is possible that the observed moderate role played by CgTpo3 in clotrimazole resistance could result from an indirect effect in the expression of *CgCDR1* or *CgCDR2*, the effect of *CgTPO3* deletion in the 51800 clinical isolate on the expression of *CgCDR1* and *CgCDR2* was assessed. The deletion of *CgTPO3* was found to have only a slight effect on *CgCDR2* expression and no effect on *CgCDR1* (**Figure 6B**). This result strongly suggests that CgTpo3 activity increases *C. glabrata* resistance to clotrimazole in clinical isolates by directly reducing its accumulation inside the cell.

## Discussion

In this work, the participation of five multidrug transporters of the MFS superfamily in the acquisition of clotrimazole resistance was evaluated in *C. glabrata* clinical isolates.

The Drug:H^+^ Antiporter family is poorly characterized in pathogenic fungi. For example, in the medically relevant pathogens *C. albicans*, *C. glabrata*, *C. parapsilosis*, *C. lusitaniae*, *C. tropicalis*, *C. guilliermondii*, *Cryptococcus neoformans*, and *Aspergillus fumigatus* there are nearly 300 ORFs predicted to encode DHA transporters, but only less than 15 have been characterized (Costa et al., 2014a). In *C. glabrata*, five transporters, CgAqr1 (Costa et al., 2013a), CgQdr2 (Costa et al., 2013b), CgTpo1_1, CgTpo1_2 (Pais et al., 2016), and CgTpo3 (Costa et al., 2014b), have been recently functionally analyzed and shown to be involved in azole drug resistance, with a stronger effect in the resistance against imidazoles. However, the impact of these findings in the clinical acquisition of resistance remained unclear.

In this work, 138 *C. glabrata* clinical isolates were screened for clotrimazole and fluconazole resistance. The majority of the isolates were characterized as susceptible-dose dependent to fluconazole, while 9 (6.5%) were identified as resistant to this drug. The relative level of resistant isolates was similar to that observed in previous studies (Sanguinetti et al., 2005; Faria-Ramos et al., 2014). On the other hand, most isolates tested (64.5%) were found to be resistant to clotrimazole. The majority of the isolates were found to be resistant to clotrimazole and susceptible-dose dependent to fluconazole. It is important to point out, however, that the identification of a very high number of clotrimazole resistant isolates is most likely due to the fact that the resistance threshold considered was that indicated for *C. albicans* (Pelletier et al., 2000), since no clotrimazole MIC breakpoint or suggested resistance threshold was ever indicated for *C. glabrata* (CLSI, 2008, 2012). Based on the fact that the fluconazole breakpoint is eightfold higher for *C. glabrata* than for *C. albicans* (CLSI, 2012), it appears reasonable to assume that the clotrimazole breakpoint for *C. glabrata* will also be higher than for *C. albicans*. Considering a similar eightfold difference, the clotrimazole breakpoint would increase to a MIC of 4, which would still imply that nearly 30% of the clinical isolates in the studied collection displays resistance toward this azole drug.

Resistance (or susceptibility) to clotrimazole and fluconazole does not seem to correlate with the body niche from which the *C. glabrata* isolates were recovered. Also, the levels of resistance to both drugs are similar whether the isolates came from blood cultures or superficial infections. This is particularly interesting, and unexpected, since fluconazole is more commonly used for the treatment of systemic infections and clotrimazole of superficial infections.

Since azoles play such an important role in clinical practice, the cross-resistance potential between imidazoles and triazoles was also addressed. Interestingly, 48 isolates (34.78%) resistant to clotrimazole were found to be, at the same time, susceptible-dose dependent to fluconazole. In agreement with the observation that there is a statistically significant correlation between the increase in CLT MIC levels and the FLC MIC levels, when considering the full 138 clinical isolates, all isolates identified as resistant to fluconazole (*n* = 9), were also found to be resistant to clotrimazole, when considering CLT resistance for MIC ≥ 1. This is consistent with the observation by Cross et al. (2000) that fluconazole-resistant bloodstream isolates of both *C. albicans* and *C. glabrata* obtained from cancer patients showed simultaneous resistance to clotrimazole and two other imidazoles: tioconazole and miconazole. These observations raise questions as to the prophylactic use of fluconazole, as it may induce not only resistance toward other triazoles (Pfaller et al., 2004, 2007, 2008), but to the more widely used imidazoles, compromising the treatment of superficial infections.

Using the results obtained from the characterization of the isolate collections referred above, the role of the DHA1 transporters CgAqr1, CgQdr2, CgTpo1_1, CgTpo1_2, and CgTpo3 in the acquisition of clotrimazole resistance in the clinical setting was evaluated. The option to consider clotrimazole R, and not fluconazole R, is due to the fact that all of the drug transporters under analysis in this study confer imidazole resistance, but only CgTpo1_1, CgTpo1_2, and CgTpo3 confer triazole resistance as well. Although it is true that fluconazole and other triazole antifungals are much more relevant in the treatment of systemic life-threatening fungal infections, imidazole antifungals such as clotrimazole are widely used in skin and mucosal infections, which are themselves a widespread problem, with high recurrence rates, and constituting an open door to the development of bloodstream infections. The expression levels of four out of five of these genes (*CgAQR1*, *CgQDR2*, *CgTPO1_1*, and *CgTPO3*) were shown to directly correlate with the increase in clotrimazole resistance, being up-regulated significantly in clotrimazole resistant isolates when compared to susceptible isolates. Interestingly, the expression of *CgQDR2* had been previously shown to be increased in a clinical isolate exhibiting a *CgPDR1* gain-of function mutation (Caudle et al., 2011). However, for the remaining DHA genes considered in this study, this is the first demonstration of up-regulation in clinical isolates, possibly because, to the best of our knowledge, there are no other studies focused on the clinical acquisition of clotrimazole resistance. Although the correlation between gene expression and clotrimazole resistance was seen to be higher for *CgCDR1*, as expected given its more prominent role in this context (Miyazaki et al., 1998; Sanglard et al., 1999; Bennett et al., 2004), the expression of the DHA genes had a correlation level similar to that of *CgCDR2*. This is particularly relevant since DHA transporters are much more poorly characterized than ABC transporters. In fact, before recent studies characterizing these *C. glabrata* DHA transporters, the only MFS transporter described as being involved in drug resistance was *C. albicans* Mdr1 (Cannon et al., 2009; Morschhauser, 2010). The involvement of this transporter in the clinical acquisition of resistance was demonstrated by the significant up-regulation of its gene expression levels in *C. albicans* clinical isolates resistant to fluconazole (Sanglard et al., 1995; White, 1997), and by an increased susceptibility toward this drug upon gene disruption, proving this transporter to mediate fluconazole resistance in *C. albicans* clinical isolates (Wirsching et al., 2000b).

Interestingly, the only gene to which no correlation could be found between the expression levels and resistance acquisition was *CgTPO1_2*. This observation was somewhat unexpected since its expression was previously observed to be up-regulated following clotrimazole exposure (Pais et al., 2016). However, this lack of correlation may be due to the fact that the expression of this gene occurs in a transient manner (Pais et al., 2016). Also, the high variability between *CgTPO1_2* expression levels in the resistant isolates, when compared to the susceptible ones, compromised the statistical significance associated to the correlation between the transcript levels in these groups of isolates and their resistance levels. Indeed it is noteworthy that the average expression levels of *CgTPO1_2* were still found to be considerably higher among resistant isolates than in susceptible ones.

Similarly to what had been done before for CaMdr1 (Wirsching et al., 2000b), the relevance of CgTpo3 was further evaluated through gene deletion in a clinical isolate, selected for showing a high level of *CgTPO3* expression. Used here as a proof-of-concept, the fact that the deletion of CgTpo3 in this clinical isolate decreases clotrimazole and fluconazole resistance to azole drugs demonstrates its importance in the clinical acquisition of azole drug resistance. It is also interesting to point out that all the tested azole resistant isolates exhibit increased expression of several of the ABC and DHA drug transporters. However, their expression profile varies from strain to strain, suggesting that several evolution paths have been selected by different strains leading to the same overall azole resistance phenotype. Indeed, it appears to be the sum of the action exerted by each of the up-regulated drug eﬄux pumps, not necessarily their individual action, that ends up building a fully resistant clinical isolate.

Altogether, this study highlights the importance of the DHA transporter family in the acquisition of azole drug resistance. Significantly, these transporters are widely spread among pathogenic fungi, but only about 5% of them have been characterized so far (Costa et al., 2014a).

## Author Contributions

CC did most of the experimental work and contributed to the writing of the manuscript. MT and AG co-conceived this work and contributed to the writing of the manuscript. JR, IM, AS-D, MC and SC-O contributed with part of the experimental work.

## Conflict of Interest Statement

The authors declare that the research was conducted in the absence of any commercial or financial relationships that could be construed as a potential conflict of interest.
